# Park use patterns and park satisfaction before and after citywide park renovations in low-income New York City neighborhoods

**DOI:** 10.1038/s41598-025-07264-3

**Published:** 2025-07-02

**Authors:** Rachel L. Thompson, Katarzyna E. Wyka, Kelly R. Evenson, Lorna E. Thorpe, Glen D. Johnson, Brian T. Pavilonis, Terry T.-K. Huang

**Affiliations:** 1 Center for Systems and Community Design, Graduate School of Public Health and Health Policy, City University of New York (CUNY), New York, NY USA; 2 NYU-CUNY Prevention Research Center, New York, NY USA; 3 Department of Environmental, Occupational, and Geospatial Health Sciences, Graduate School of Public Health and Health Policy, City University of New York (CUNY), New York, NY USA; 4 Department of Epidemiology, Gillings School of Global Public Health, University of North Carolina–Chapel Hill, Chapel Hill, NC USA; 5 Department of Population Health, Grossman School of Medicine, New York University (NYU), New York, NY USA

**Keywords:** Park renovation, Park use, Park satisfaction, Urban health, Built environment, Natural experiment, Epidemiology, Health policy, Psychology and behaviour, Urban ecology

## Abstract

Urban parks may promote health through physical activity, stress management, and social connectedness. However, poor-quality parks in disrepair are underutilized, limiting these benefits. This study evaluated the impact of a citywide park renovation program known as the Community Parks Initiative (CPI) on changes in park use patterns and park satisfaction among residents living in low-income New York City neighborhoods. Repeated cross-sectional surveys were administered to residents living near 31 parks undergoing CPI renovations (545 pre-renovation and 201 post-renovation respondents) and near 21 parks in socio-demographically matched control neighborhoods (345 pre-renovation and 129 post-renovation respondents). Surveys measured self-reported past-month park visits, typical park visit duration, and satisfaction with park quality and facilities. Using a difference-in-differences (DID) approach, generalized estimating equations were fit to compare changes over time in park use and satisfaction among residents living near parks receiving renovations compared to those living near control parks. Models were adjusted for age, body mass index, income, public housing, marital status, and children in household. Residents in neighborhoods receiving park renovations reported a larger increase in minutes spent at the park on weekdays [DID = 30.0 min (95% CI 10.3, 49.7)] and total minutes spent at the park in the last 30 days [DID = 466.3 min (95% CI 63.0, 869.6)] compared to controls. Residents of renovated park neighborhoods also reported larger increases in park satisfaction relative to residents of control neighborhoods, with the largest improvements in the percent of residents satisfied with overall park quality [DID = 38.4% (95% CI 25.2, 51.6)] and maintenance of grounds and facilities [DID = 40.9% (95% CI 27.7, 54.1)]. This study provides evidence that park renovations are an important urban planning strategy to support community health through increased park use and improved park perceptions.

**Supplementary Information:**

The online version contains supplementary material available at 10.1038/s41598-025-07264-3.

## Introduction

The twenty-first century has experienced a significant shift towards urban living, with over 55% of the world’s population now residing in urban areas, a number projected to rise to 68% by 2050^1^. Urbanization brings with it a host of health challenges. The World Health Organization cites physical inactivity as a top urban health concern due to its link to obesity, diabetes, and other chronic diseases, particularly in urban settings lacking infrastructure for active living^2^. Urbanization has also been linked to high rates of depression, anxiety, and poor mental health, effects which are more pronounced among disadvantaged low-income and minority groups^2,3^.

Amidst the pressing urban health challenges of the twenty-first century, greenspaces serve as “lungs of the city” promoting both mental and physical health through various pathways. Environmentally, green spaces contribute to improved air quality and reduced urban heat islands, improving respiratory health and overall comfort^4–6^. Socially, parks provide communal spaces that foster social interactions and community cohesion, supporting well-being and potentially reducing feelings of isolation^7,8^. The physical spaces of parks may encourage outdoor activities such as walking, jogging, and recreational sports by providing free and publicly-accessible areas for health-promoting physical activity behaviors^9^. Moreover, the natural peaceful and aesthetic landscapes of urban greenspaces can provide mental health benefits, including reduced stress and improved mood^10^.

Many of the health benefits associated with urban parks and greenspaces are likely contingent upon them being used. High-quality parks that are well-maintained and perceived as safe are used more frequently than those in disrepair or with incivilities^11–14^. We previously demonstrated that frequent use of high-quality, recently renovated urban parks in low-income neighborhoods was associated with higher physical activity^15^ and lower stress^16^. These findings suggest that park quality may be a precondition to the benefits of frequent park use on health outcomes. We also previously showed that positive perceptions of park spaces are as important as park use in the relationship between urban parks and quality of life^17^. Disparities in access to high-quality urban park spaces contribute to health disparities in urban areas, where low-income neighborhoods have access to lower-quality facilities and less greenspace overall compared to high-income neighborhoods^18–20^.

The implementation of park renovations in low-income urban areas may be one means of addressing health disparities through simultaneously improving access to and use of high-quality urban park spaces. Numerous studies have demonstrated that park renovation interventions in urban areas have been associated with an increase in the number of observed park users^21–24^. However, existing research has largely focused on small-scale interventions (< 10 park sites) and relied on systematic observations or intercept surveys of park visitors to measure park use^21^. These methodologies, while valuable, primarily capture park-level or visitor-level changes in park use and have provided us with limited understanding of how park improvements might affect the park use patterns of neighborhood residents living near intervention parks. Furthermore, despite extensive literature on the determinants of park satisfaction^14,25–28^, there is a notable lack of robust intervention studies evaluating how park renovations impact park satisfaction, particularly at the neighborhood level.

This study seeks to address these gaps by evaluating the Community Parks Initiative (CPI), a citywide park renovation program in low-income New York City (NYC) neighborhoods, and its effects on neighborhood-level self-reported park use and satisfaction through one of the largest quasi-experimental studies of park renovations to date. Since its inception in 2014, the CPI has led to the renovation of over 60 parks, resulting in a vast array of infrastructure improvements including: increased greening and shading, improved ball courts and walking paths, upgraded playground equipment, and new installation of adult fitness equipment^29^. A large number of parks were renovated between 2017 and 2021, which presented a unique opportunity to study the effects of park renovations on changes in park use patterns, park perceptions, and health among residents living near these parks^30^. Using repeated cross-sectional survey data sampled from residents in the neighborhoods surrounding renovated parks and matched control parks, this study aimed to better understand and quantify how the CPI impacted neighborhood-level satisfaction with improved park spaces and patterns of self-reported park use. By comparing these outcomes before and after the implementation of renovations, this study explored how large-scale park improvements influenced park-related behaviors and perceptions within the broader communities served by renovated parks.

## Methods

### Study design and setting

The Physical Activity and Redesigned Community Spaces (PARCS) study was a quasi-experimental study that sought to evaluate the effects of CPI park renovations on health and behavioral changes among low-income NYC communities^30^. The study took place between 2016 and 2022, and collected a variety of park use, physical activity, and self-reported health status measures from adult residents living within walking distance (0.3-mile radius or about five city blocks) of a public park eligible for renovation.

The present analysis included survey responses from residents living in neighborhoods surrounding 31 parks that underwent redesign and renovation as part of the CPI (intervention parks), as well as survey responses from residents in socio-demographically matched control neighborhoods surrounding 21 parks that did not receive renovations (control parks). Park sites were considered eligible for the study if they were located in neighborhoods with two of the following characteristics: high poverty (≥ 20% of residents living below the federal poverty line), rapid population growth (≥ 25% growth from 2000 to 2010) and high population density (≥ 110 residents per acre of land). Additionally, eligible parks must not have received more than $250,000 in capital investments during the previous 20 years. Matching of intervention and control sites was done using best frequency matches (± 6% difference) across aggregated neighborhood-level demographic characteristics, including percent of the population ≥ 18y, percent White residents, percent Black residents, percent Asian residents, percent Latino/a residents, percent change in population between 2000 and 2010, and percent of residents living below the federal poverty line^30^.

### Intervention

The intervention involved a comprehensive redesign and renovation of intervention parks as a part of the CPI park quality improvement program. Both control and intervention park sites were considered eligible for CPI renovations, but only intervention park sites were scheduled to be renovated during the study period. Renovations at intervention sites were implemented by NYC Parks, with initial designs created based on input from neighborhood residents^31^. The renovations aimed to improve the quality, accessibility, and inclusivity of parks by incorporating features appealing to diverse demographic groups^31^. While renovations varied depending on specific neighborhood inputs and park site conditions, common renovated features included: aesthetic improvements, greenery and shade cover, seating areas, improved play equipment, renovated sports facilities, comfort stations, and enhanced accessibility^22^.

### Survey data collection

Recruitment and survey data collection methods for the PARCS study have been previously described in detail^30,32^. The study adopted a variety of sampling techniques, including park-level audits using the System for Observing Play and Recreation in Communities (SOPARC)^22^, cross-sectional survey sampling of neighborhoods, and the enrollment of a longitudinal prospective cohort^30^. In this paper, we report on changes in park use and satisfaction at the neighborhood-level, collected through independent cross-sectional survey samples collected from participating communities before and after CPI park renovations were implemented. Adult residents (≥ 18 y) living within a 0.3-mile radius of a study park with no mobility issues that spoke English, Spanish, or Chinese (most common languages in NYC) were considered eligible to participate in the study. We used a multifaceted and active approach to recruitment, leveraging the existing infrastructure and activities of the NYC Parks community outreach organization *Partnership for Parks*^30^*.* Over the course of the study, designated study ambassadors built a professional and trusted presence in the community through regular engagement events in study neighborhoods, at study parks, and on social media^32^. Recruitment efforts targeted residents in both public and non-public housing, given that park sites were purposefully selected from low-income neighborhoods that had a high percentage of residents living in NYC Housing Authority (NYCHA) public housing sites^30^. When deemed eligible by study staff, residents filled out a survey on their mobile phone using a survey app or on paper if preferred by the participant. Participants were given $50–75 incentives for completing surveys. Recruitment and data collection was completed in two waves: pre-renovation and post-renovation. Pre-renovation survey responses were collected from a sample of eligible residents prior to the implementation of CPI renovations at intervention parks (2016–2018), after which intervention parks were closed for renovation and gradually reopened between 2017 and 2021. A new set (post-renovation) of survey responses was collected from a new unique sample of eligible residents after the implementation of renovations and reopening of intervention parks (2018–2022).

### Measures

#### Park use outcomes

Three questions were used to capture different elements of past-month study park use, including frequency and duration of study park use, which were adapted from a previously validated survey instrument demonstrating excellent test–retest reliability (3/3 questions with intra-class correlation > 0.8)^33,34^. The original survey questions assessed study park use “In the past 3 months”^33^ but were modified for our study to “In the past 30 days” to better align with our study’s planned data collection timeline. The first question asked residents to provide an estimate of past-month park use frequency: “In the past 30 days, on average, how often have you visited [study park name]?” with options (1) daily, (2) 4–6 times per week, (3) 2–3 times per week, (4) once per week, (5) 2–3 times per month, (6) once per month, (7) less than once per month, and (8) have not visited in the past 30 days. For ease of interpretation, this scale was converted to the number of days the resident visited the study park in the past month by taking each response’s midpoint (as described in a previously published protocol)^35^. For example, those who reported daily park use were assigned a value of 30 days, those who reported visiting 4–6 times per week were assigned a value of 20 days, those who reported visiting once per week were assigned a value of 4 days, etc.

The second and third questions relating to past-month park use asked residents to provide an estimate of the usual length of time they spent at the park on weekdays and weekend days. These questions were worded as follows: “In the past 30 days, how long did you usually spend at [study park name] on [weekdays/weekend days]?” with options (1) < 30 min, (2) 30–59 min, (3) 1– < 2 h, (4) 2- < 3 h, (5) 3– < 4 h, (6) 4+ hours, and (7) not applicable. For ease of interpretation, this scale was also converted to the number of minutes the resident usually visited the study park in the past month by taking each response’s midpoint. For example, those who reported < 30 min of study park use were assigned a value of 15 min, those who reported 30–59 min of study park use were assigned a value of 45 min, those who reported 4+ hours of study park use were assigned a value of 240 min, etc. Those who responded with “not applicable” were assigned a value of 0 min.

Four additional past-month park use outcome variables were created from responses to these three questions. The total minutes each resident spent at the study park in the past month was found by multiplying the past-month frequency of study park use (in days) by the average past-month duration of study park use across weekdays and weekend days (in minutes). Additionally, a binary variable was created to indicate any past-month park visits, categorized as either “ ≥ once per month” or “ < once per month.” Similarly, typical visits lasting 30 min or longer on weekends or weekdays were recorded as binary variables, with the categories “ ≥ 30 min on a [weekday/weekend day]” and “ < 30 min on a [weekday/weekend day].”

#### Park satisfaction outcomes

Five questions in the survey assessed residents’ satisfaction with study park quality and facilities, which were adapted from a previously validated survey instrument with good test–retest reliability (4/5 questions with intra-class correlation ≥ 0.4)^33^. Individuals were asked to rate their agreement with the following statements regarding their study park using a six-category Likert scale: “I am satisfied with the overall quality”, “I am satisfied with the facilities available”, “I am satisfied with the playground”, “I am satisfied with the walking/cycling tracks”, and “I am satisfied with the maintenance of the grounds and facilities”. The response options included: (1) strongly disagree, (2) disagree, (3) neither agree nor disagree, (4) agree, (5) strongly agree, and (6) don’t know. Each question was subsequently converted into a binary variable indicating either “satisfied” or “not satisfied”. The “satisfied” category included those residents who replied “agree” or “strongly agree” to the respective satisfaction statement, while the “not satisfied” category included all other responses.

#### Sociodemographic variables

Sociodemographic variables included sex (male, female), age, body mass index (BMI), race/ethnicity (Latino/a, non-Latino/a Black, other or multiracial), annual household income ($20,000 or more, less than $20,000), education (high school graduate or less, some college or more), employment status (employed or self-employed, not employed), public housing (non-NYCHA resident, NYCHA resident), marital status (never married, married, divorced/separated/widowed), and children in household (no children, one or more children). For race/ethnicity, the other or multiracial group included non-Latino/a White and any other race, and for employment, the not employed group included residents who were homemakers, students, retired, unemployed, or unable to work. Age and BMI (kg/m^2^) were analyzed continuously and also separated into three groups: 18–34 y, 35–49 y and 50–78 y for age, and healthy (BMI < 25 kg/m^2^), overweight (BMI 25–29 kg/m^2^), and obese (BMI ≥ 30 kg/m^2^) for BMI.

### Statistical analysis

Residents who completed at least one survey question on park use and at least one question on park satisfaction were included in this analysis. Multiple imputation by chained equations was used to impute a small number of missing outcomes and sociodemographic variables. Missingness across variables ranged from 0.2% missing (minutes spent at the study park on weekdays) to 7.9% missing (annual household income). Twenty-five imputed datasets were generated from the original data using the full set of outcome variables and sociodemographic variables as predictors. All statistical analyses were pooled across the 25 imputed data sets. A summary of imputed sociodemographic characteristics is available in Supplementary Table 1.

We produced descriptive statistics (n (%), mean (SD)) for all sociodemographic variables in the pre- and post-renovation samples, stratified by intervention group. Chi-squared tests and t-tests screened for potential differences between the pre- and post-renovation survey samples. We estimated the mean (for continuous variables) or percent (for binary variables) and SE for all outcomes in both survey samples, and stratified by intervention group, as the pooled mean/percent (SE) across 25 imputed datasets.

We used a difference-in-differences (DID) approach to compare changes over time in park use and park satisfaction measures among residents from renovated park neighborhoods versus control park neighborhoods. Linear generalized estimating equations (GEE) were used to account for autocorrelation within park site clusters. The models included variables for time (pre-renovation vs. post-renovation), intervention group (renovated vs. control parks), and an interaction between time and intervention group (the DID estimator). While none of the outcome variables themselves were Gaussian-distributed, it has been shown that linear models are suitable for DID analysis with non-normal and binary outcomes, because the outcome of interest modeled by the DID estimator is *change* in the outcome (which should be approximately normally distributed and therefore not likely to produce residual distributions violating the assumptions of linear models)^36,37^. Additionally, using linear GEE models allowed for the interpretation of DID estimators on the absolute difference scale, which is more intuitive when assessing differential change over time compared to estimates on a multiplicative difference scale.

Stratified DID estimates for certain sociodemographic variables were also explored by fitting three-way interaction terms between time, intervention group, and a given sociodemographic variable. We screened for significant (*p* < 0.1) three-way interactions with sex, age category, BMI category, race/ethnicity, annual household income, education, employment status, public housing, marital status, and children in household. Stratified DID estimates are shown for any sociodemographic variable that had a statistically significant three-way interaction with any park use or park satisfaction outcome variable.

DID estimators were reported both unadjusted and adjusted for sociodemographic variables found to be imbalanced between the intervention and control groups in either the pre-renovation or post-renovation survey sample. For all outcomes, we present the GEE model-estimated change in the mean outcome post-renovation minus pre-renovation for the intervention and control groups separately, with 95% confidence intervals. P-values for DID estimators were corrected using the Benjamini-Hochberg (BH) procedure to control for the false discovery rate and maintain a two-tailed α = 0.05^38^. *P*-values for stratified DID estimates were left uncorrected, as we considered these results to be exploratory.

Most (88%, n = 289) post-renovation survey responses were recorded before the COVID-19 pandemic began in March 2020, while 12% (n = 41) did so afterward. Since the COVID-19 pandemic may have differentially impacted the park use patterns of residents who completed surveys during and after March 2020, we performed a sensitivity analysis excluding these 41 observations to compare with the full sample results.

Data cleaning and analyses were performed in R software version 4.4.1 (https://www.R-project.org). Multiple imputation and pooling of GEE model estimates were completed with the *mice* package^39^. GEE models were fit using the *geepack* package^40^. Contrasts were extracted from fitted interaction models using the *emmeans* package^41^. Data visualizations were created using *ggplot2*^42^. For statistical tests, significant results were defined as *p* < 0.05, and marginally significant results were defined as 0.05 < *p* < 0.1.

## Results

### Sociodemographic characteristics of residents living near study parks

Sociodemographic characteristics of sampled residents are provided in Table 1. A total of 1,220 unique individual residents living within 0.3 miles of a study park completed park use and satisfaction surveys. This included 746 adults living near 31 parks undergoing CPI renovations (545 pre-renovation and 201 post-renovation respondents) and 474 adults living near 21 control parks in sociodemographically-matched neighborhoods (345 pre-renovation and 129 post-renovation respondents). The average number of respondents per park site was 17.1 in the pre-renovation sample and 6.3 in the post-renovation sample.

**Table 1:** Sociodemographic characteristics of sampled residents living within 0.3 miles of an intervention or control park, pre- and post-renovation.

	Overall Sample			Pre-Renovation by Group			Post-Renovation by Group		
	Pre-Renovation N = 890^*a*^	Post-Renovation N = 330^*a*^	p-value^*b*^	Intervention N = 545^*a*^	Control N = 345^*a*^	p-value^*b*^	Intervention N = 201^*a*^	Control N = 129^*a*^	p-value^*b*^
Sex			0.300			0.120			0.400
Female	715 (81%)	267 (83%)		428 (79%)	287 (83%)		159 (82%)	108 (86%)	
Male	172 (19%)	53 (17%)		114 (21%)	58 (17%)		35 (18%)	18 (14%)	
(Missing)	3	10		3	0		7	3	
Age	38 (12)	41 (13)	** < 0.001**	38 (13)	38 (11)	0.600	41 (12)	42 (14)	0.800
Age Category			**0.002**			0.500			**0.070**
18-34y	394 (45%)	104 (34%)		241 (45%)	153 (45%)		59 (32%)	45 (37%)	
35-49y	305 (35%)	125 (41%)		179 (34%)	126 (37%)		85 (46%)	40 (33%)	
50-78y	172 (20%)	79 (26%)		110 (21%)	62 (18%)		42 (23%)	37 (30%)	
(Missing)	19	22		15	4		15	7	
Body Mass Index (BMI)	30 (7)	30 (8)	> 0.900	29 (7)	30 (7)	**0.064**	30 (8)	30 (7)	0.800
BMI Category			**0.077**			**0.060**			0.400
Healthy (BMI < 25 kg/m^2^)	206 (24%)	89 (30%)		132 (26%)	74 (22%)		58 (33%)	31 (26%)	
Overweight (BMI 25–29 kg/m^2^)	269 (32%)	78 (27%)		170 (33%)	99 (29%)		43 (25%)	35 (30%)	
Obese (BMI ≥ 30 kg/m^2^)	376 (44%)	125 (43%)		209 (41%)	167 (49%)		73 (42%)	52 (44%)	
(Missing)	39	38		34	5		27	11	
Race/Ethnicity			0.400			> 0.900			0.500
Latino/a	408 (47%)	130 (42%)		247 (47%)	161 (47%)		74 (40%)	56 (46%)	
Non-Latino/a Black	326 (37%)	123 (40%)		198 (38%)	128 (37%)		79 (43%)	44 (36%)	
Other or multiracial	136 (16%)	53 (17%)		83 (16%)	53 (15%)		32 (17%)	21 (17%)	
(Missing)	20	24		17	3		16	8	
Annual Household Income			0.300			0.300			**0.082**
$20,000 or more	410 (49%)	131 (45%)		258 (50%)	152 (47%)		85 (50%)	46 (39%)	
Less than $20,000	426 (51%)	157 (55%)		253 (50%)	173 (53%)		86 (50%)	71 (61%)	
(Missing)	54	42		34	20		30	12	
Education			0.700			0.500			0.800
High school graduate or less	432 (50%)	134 (49%)		266 (51%)	166 (48%)		76 (48%)	58 (50%)	
Some college or more	435 (50%)	142 (51%)		258 (49%)	177 (52%)		83 (52%)	59 (50%)	
(Missing)	23	54		21	2		42	12	
Employment Status			0.300			0.200			0.140
Employed or self-employed	453 (51%)	157 (48%)		268 (50%)	185 (54%)		102 (51%)	55 (43%)	
Not employed	429 (49%)	172 (52%)		272 (50%)	157 (46%)		98 (49%)	74 (57%)	
(Missing)	8	1		5	3		1	0	
Public Housing			0.600			** < 0.001**			**0.049**
Non-NYCHA resident	447 (50%)	157 (49%)		299 (55%)	148 (43%)		104 (53%)	53 (42%)	
NYCHA resident	440 (50%)	164 (51%)		243 (45%)	197 (57%)		91 (47%)	73 (58%)	
(Missing)	3	9		3	0		6	3	
Marital Status			0.600			**0.032**			0.900
Never married	446 (50%)	175 (53%)		283 (52%)	163 (48%)		107 (54%)	68 (53%)	
Married	265 (30%)	89 (27%)		145 (27%)	120 (35%)		55 (28%)	34 (26%)	
Divorced, separated, or widowed	173 (20%)	64 (20%)		113 (21%)	60 (17%)		37 (19%)	27 (21%)	
(Missing)	6	2		4	2		2	0	
Children in Household			0.400			**0.002**			0.400
No children	190 (22%)	79 (24%)		135 (25%)	55 (16%)		45 (22%)	34 (27%)	
One or more children	686 (78%)	250 (76%)		402 (75%)	284 (84%)		156 (78%)	94 (73%)	
(Missing)	14	1		8	6		0	1	

*BMI* body mass index, *NYCHA* New York City housing authority, *SD* standard deviation.

^a^n (%); Mean (SD).

^b^Pearson’s Chi-squared test; Welch Two Sample t-test; significant (*p* < 0.05) and marginally significant (0.05 < *p* < 0.1) *p*-values are bolded.

In the overall sample, pre-renovation and post-renovation surveyed residents were similar in most sociodemographic characteristics, with the exception of age (Table 1). The post-renovation sample was slightly older on average than the pre-renovation sample (mean age post-renovation (SD) = 41 (13); mean age pre-renovation (SD) = 38 (12); *p* < 0.001). Surveyed residents were predominantly female (pre-renovation 81%; post-renovation 83%), overweight or obese (pre-renovation 76%; post-renovation 70%), and either Latino/a (pre-renovation 47%; post-renovation 42%) or non-Latino/a Black (pre-renovation 37%; post-renovation 40%). Approximately half of the residents reported an annual household income of less than $20,000 (pre-renovation 51%; post-renovation 55%), a high school-level education or less (pre-renovation 50%; post-renovation 49%), being employed or self-employed (pre-renovation 51%; post-renovation 48%), and residence in public housing (pre-renovation 50%; post-renovation 51%). Approximately half of the surveyed residents were never married (pre-renovation 50%; post-renovation 53%), and a majority reported having one or more children in their household (pre-renovation 78%; post-renovation 76%).

In both the pre-renovation and post-renovation samples, residents from intervention and control neighborhoods also exhibited similar sociodemographic characteristics, with small differences in age, BMI, annual household income, public housing status, marital status, and the presence of children in the household (Table 1). In the pre-renovation sample, the intervention group had a significantly lower proportion of residents living in public housing (45% vs. 57%; *p* < 0.001), and a larger proportion of residents who had never married (52% vs. 48%; *p* = 0.032) and had no children (25% vs. 16%; *p* = 0.002) compared to the control group. In the post-renovation sample, the intervention group also had a significantly lower proportion of residents living in public housing (47% vs. 58%; *p* = 0.049) compared to the control group. There were marginally significant differences in the composition of the intervention and control samples by BMI category and continuous BMI (pre-renovation only), age category (post-renovation only), and annual household income (post-renovation only). In the results that follow, covariate-adjusted models refer to GEE models that were adjusted for these imbalanced covariates (i.e., age category, BMI category, annual household income, public housing, marital status, and children in household).

### Changes in past-month park use

**Fig. 1:**
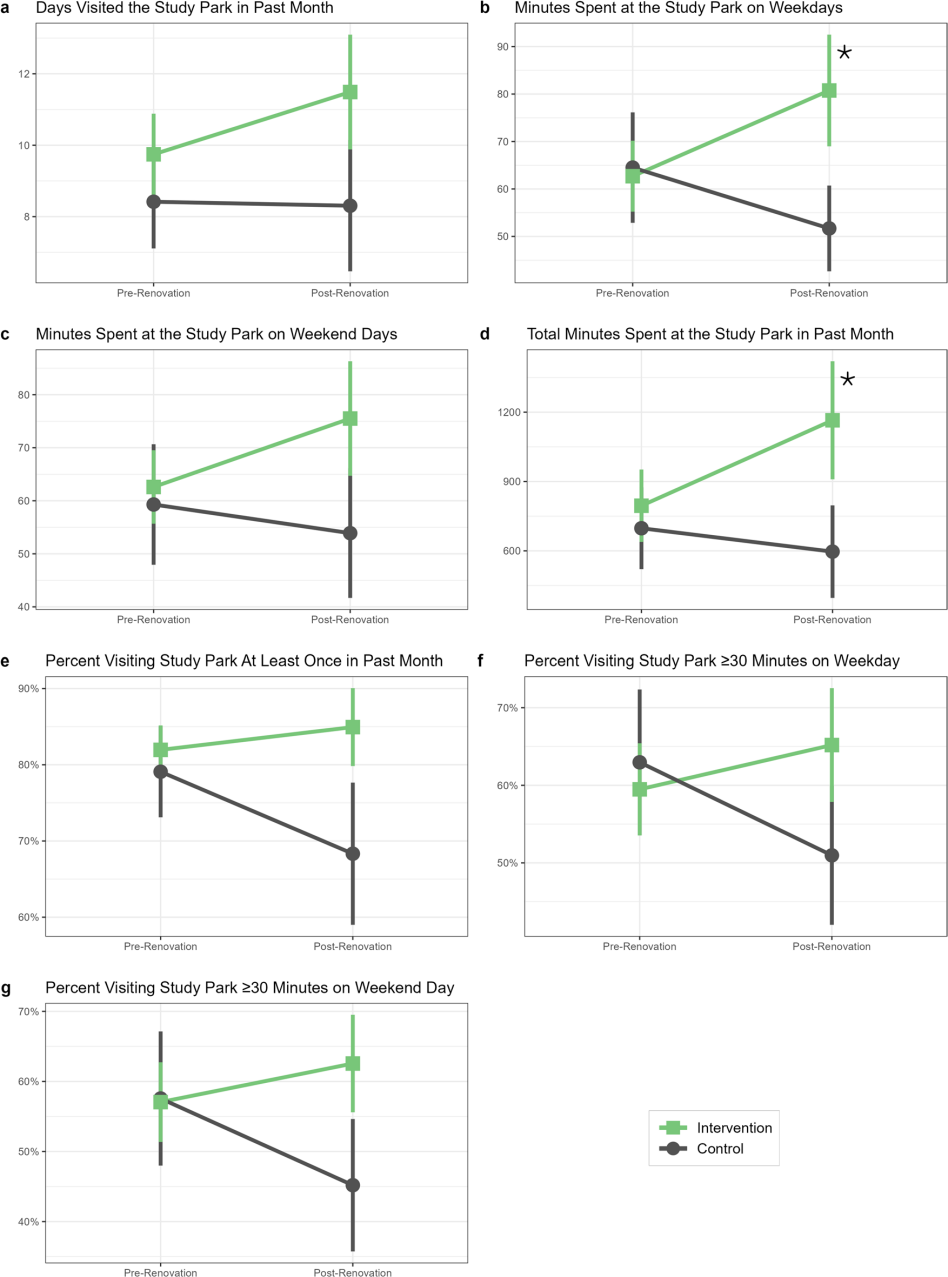
Mean park use measures among adult residents living near study parks before and after CPI park renovations. Means by intervention group (green square = intervention, grey circle = control) and 95% confidence intervals (vertical bars) were estimated using linear GEE models that included variables for time (pre-renovation vs. post-renovation), intervention group (renovated vs. control parks), and an interaction between time and intervention group (the DID estimator). An asterisk denotes a significant (*p* < 0.05) DID estimator after adjusting for age group, BMI category, annual household income, public housing, marital status, and children in household. *BMI* body mass index, *CPI* community parks initiative, *DID* difference-in-differences, *GEE* generalized estimating equations.

As shown in Table 2 and Fig. 1, residents in neighborhoods receiving CPI park renovations had a larger increase in minutes spent at the park on weekdays [covariate-adjusted DID = 30.0 min (95% CI 10.3, 49.7); BH-corrected *p* = 0.006] and total minutes spent at the park in the past month [covariate-adjusted DID = 466.3 min (95% CI 63.0, 869.6); BH-corrected p = 0.040] than residents in control neighborhoods. There was no difference between intervention and control neighborhoods in the change in number of days of the past month that residents reported visiting the study park [covariate-adjusted DID = 1.8 days (95% CI − 1.2, 4.9); BH-corrected *p* = 0.240] or minutes residents usually spent at the park on weekend days [covariate-adjusted DID = 16.7 min (95% CI − 3.7, 37.1); BH-corrected *p* = 0.119].

**Table 2:** Changes in self-reported past-month study park use among adult residents living in intervention versus control park neighborhoods.

Past-month study park use	Intervention park neighborhoods			Control park neighborhoods			Difference-in-differences		
	Pre-renovation^a^ (n = 545)	Post-renovation^a^ (n = 201)	Change (95% CI)^b^	Pre-renovation^a^ (n = 345)	Post-renovation^a^ (n = 129)	Change (95% CI)^b^	Unadjusted DID estimator (95% CI)^c^	Adjusted DID Estimator (95% CI)^c,d^	*p*-value for adjusted DID estimator^e^
Days visited	9.7 (0.6)	11.5 (0.8)	1.7 (− 0.2, 3.7)	8.4 (0.7)	8.3 (0.9)	− 0.1 (− 2.4, 2.1)	1.9 (− 1.1, 4.9)	1.8 (− 1.2, 4.9)	0.240
Minutes spent at park on weekdays	62.7 (3.8)	80.8 (6.0)	18.1 (4.1, 32.0)	64.5 (5.9)	51.7 (4.6)	− 12.8 (− 27.6, 1.9)	30.9 (10.6, 51.2)	30.0 (10.3, 49.7)	**0.006**
Minutes spent at park on weekend days	62.6 (3.5)	75.5 (5.5)	12.9 (0.1, 25.7)	59.3 (5.8)	53.9 (6.2)	− 5.4 (− 22.1, 11.3)	18.3 (− 2.8, 39.4)	16.7 (− 3.7, 37.1)	0.119
Total minutes spent at park	795.2 (79.9)	1164.9 (130.3)	369.6 (69.8, 669.5)	697.8 (90.3)	596.6 (102.2)	− 101.2 (− 368.6, 166.3)	470.8 (69.1, 872.6)	466.3 (63.0, 869.6)	**0.040**
Percent visiting park ≥ once per month	82.0 (1.6)	84.9 (2.6)	3.0 (− 3.0, 9.0)	79.1 (3.0)	68.3 (4.8)	− 10.8 (− 21.8, 0.3)	13.7 (1.1, 26.4)	13.0 (0.2, 25.8)	**0.057**
Percent visiting park ≥ 30 min on a weekday	59.5 (3.0)	65.2 (3.7)	5.7 (− 3.7, 15.2)	63.0 (4.8)	51.0 (4.6)	− 12.0 (− 25.0, 1.0)	17.7 (1.6, 33.8)	16.1 (1.0, 31.2)	**0.051**
Percent visiting park ≥ 30 min on a weekend day	57.1 (2.9)	62.6 (3.6)	5.5 (− 3.5, 14.5)	57.6 (4.9)	45.2 (4.8)	− 12.4 (− 25.8, 1.1)	17.9 (1.7, 34.1)	16.4 (0.9, 32.0)	**0.051**

All estimates presented in table were pooled across GEE models fit to 25 imputed data sets. All GEE models included variables for time (pre-renovation vs. post-renovation), intervention group (renovated vs. control parks), and an interaction between time and intervention group (the DID estimator).

*BMI* body mass index, *CI* confidence interval, *DID* difference-in-differences, *GEE* generalized estimating equations, *SE* standard error.

^a^Unadjusted mean (SE) or percent (SE).

^b^Unadjusted within-group change (post–pre).

^c^Difference in change in the given outcome measure in the intervention group minus the control group, as estimated by the interaction term between time * intervention group.

^d^Adjusted for age group, BMI category, annual household income, public housing, marital status, and children in household.

^e^Corrected using the Benjamini–Hochberg procedure to control the false discovery rate; significant (*p* < 0.05) and marginally significant (0.05 < *p* < 0.1) *p*-values are bolded.

Compared to control neighborhoods, intervention neighborhoods experienced a slightly larger increase in the percent of residents that visited the study park once or more in the past month [covariate-adjusted DID = 13.0%, (95% CI 0.2, 25.8); BH-corrected p = 0.057], visited the study park for 30 min or longer on a weekday [covariate-adjusted DID = 16.1%, (95% CI 1.0, 31.2); BH-corrected *p* = 0.051], and visited the study park for 30 min or longer on a weekend day [covariate-adjusted DID = 16.4% (95% CI 0.9, 32.0); BH-corrected *p* = 0.051], although these differences were only marginally significant (Table 2, Fig. 1).

### Changes in past-month park use stratified by sociodemographics

Table 3 demonstrates the varying effects of park renovation on changes in past-month park use observed across different sociodemographic groups, including: BMI, race/ethnicity, annual household income, employment status, and the presence of children in households. We found no differences in the effects of park renovation on changes in past-month park use by sex, age, education, public housing, or marital status.

**Table 3:** Park use DID estimates stratified by BMI category, race/ethnicity, annual household income, employment status, and children in household.

	Days visited		Minutes spent on weekdays		Minutes spent on weekends		Total minutes at park	
	Adjusted DID estimator (95% CI)^a,b^	*p*-value^c^	Adjusted DID estimator (95% CI)^a,b^	*p*-value^c^	Adjusted DID estimator (95% CI)^a,b^	*p*-value^c^	Adjusted DID estimator (95% CI)^a,b^	*p*-value^c^
BMI category								
Healthy (BMI < 25 kg/m^2^)	− 0.8 (− 5.5, 3.9)	0.733	38.2 (4.1, 72.3)	**0.028**	7.2 (− 34.0, 48.4)	0.731	118.5 (− 553.3, 790.2)	0.729
Overweight (BMI 25–29 kg/m^2^)	6.2 (1.2, 11.2)	**0.014**	34.4 (− 0.8, 69.7)	0.055	7.5 (− 26.4, 41.4)	0.666	576.0 (− 73.6, 1225.7)	0.082
Obese (BMI ≥ 30 kg/m^2^)	0.4 (− 4.0, 4.8)	0.865	20.8 (− 8.6, 50.1)	0.165	28.0 (− 1.5, 57.4)	0.063	579.2 (− 46.2, 1204.7)	0.069
Race/ethnicity								
Latino/a	2.5 (− 1.5, 6.4)	0.224	37.5 (10.1, 65.0)	**0.007**	27.2 (− 3.4, 57.7)	0.081	694.4 (77.5, 1311.2)	**0.027**
Non-Latino/a black	− 0.4 (− 5.3, 4.5)	0.879	28.8 (− 0.2, 57.9)	0.052	12.9 (− 13.8, 39.5)	0.344	377.7 (− 262.1, 1017.6)	0.247
Other/multiracial	4.7 (− 0.9, 10.2)	0.100	8.5 (− 27.7, 44.8)	0.644	− 0.5 (− 43.2, 42.2)	0.981	53.0 (− 577.0, 682.9)	0.869
Annual household income								
$20,000 or more	1.1 (− 3.1, 5.2)	0.616	41.9 (16.9, 66.8)	**0.001**	33.3 (5.2, 61.5)	**0.020**	573.0 (69.3, 1076.7)	**0.026**
Less than $20,000	2.0 (− 1.9, 5.9)	0.313	19.5 (− 6.2, 45.2)	0.137	1.3 (− 25.3, 27.8)	0.924	346.4 (− 219.9, 912.7)	0.230
Employment status								
Employed or self-employed	1.1 (− 2.9, 5.2)	0.580	15.1 (− 10.6, 40.8)	0.249	− 3.2 (− 30.7, 24.3)	0.817	80.6 (− 452.6, 613.7)	0.767
Not employed	2.3 (− 2.0, 6.6)	0.301	41.6 (15.5, 67.6)	**0.002**	29.8 (3.5, 56.2)	**0.026**	791.7 (213.3, 1370.2)	**0.007**
Children in household								
No children	− 0.2 (− 6.5, 6.2)	0.963	5.7 (− 25.6, 36.9)	0.722	15.9 (− 16.4, 48.2)	0.333	371.0 (− 340.5, 1082.5)	0.306
One or more children	2.4 (− 0.9, 5.6)	0.158	36.8 (13.6, 60.0)	**0.002**	16.5 (− 7.8, 40.8)	0.184	498.8 (41.3, 956.4)	**0.033**

All estimates presented in table were pooled across GEE models fit to 25 imputed data sets. All GEE models included variables for time (pre-renovation vs. post-renovation), intervention group (renovated vs. control parks), and an interaction between time and intervention group (the DID estimator).

*BMI* body mass index, *CI* confidence interval, *DID* difference-in-differences, *GEE* generalized estimating equations.

^a^Difference in change in the given outcome measure in the intervention group minus the control group, as estimated by the interaction term between time * intervention group.

^b^Adjusted for age group, BMI category, annual household income, public housing, marital status, and children in household.

^c^Raw (uncorrected) *p*-value; significant (*p* < 0.05) *p*-values are bolded.

#### BMI

Across all sociodemographic groups, a significant effect of park renovation on the number of days residents reported visited the study park in the past month was found only among residents classified with overweight BMI (25–29 kg/m^2^) [covariate-adjusted DID = 6.2 days (95% CI 1.2, 11.2); *p* = 0.014] (Table 3). Across the BMI categories, only individuals with a healthy BMI (< 25 kg/m^2^) experienced a significant effect of park renovation on minutes spent at the study park on weekdays [covariate-adjusted DID = 38.2 min (95% CI 4.1, 72.3); *p* = 0.028].

#### Race/ethnicity

Latino/as experienced a larger increase in minutes spent at the park on weekdays as a result of park renovation [covariate-adjusted DID = 37.5 min (95% CI 10.1, 65.0); *p* = 0.007] compared to non-Latino/a Blacks [covariate-adjusted DID = 28.8 min (95% CI − 0.2, 57.9); *p* = 0.052] and other/multiracial residents [covariate-adjusted DID = 8.5 min (95% CI − 27.7, 44.8); *p* = 0.644] (Table 3). Latino/as also experienced a larger increase in total minutes spent at the park in the past 30 days [covariate-adjusted DID = 694.4 min (95% CI 77.5, 1311.2); *p* = 0.027] than the other two groups [non-Latino/a Black DID = 377.7 min (95% CI − 262.1, 1017.6); *p* = 0.247; other/multiracial DID = 53.0 min (95% CI − 577.0, 682.9); *p* = 0.869].

#### Annual household income

Residents who reported an annual household income of $20,000 or more experienced a significant increase in minutes spent at the park on weekdays [covariate-adjusted DID = 41.9 min (95% CI 16.9, 66.8); *p* = 0.001], minutes spent at the park on weekends [covariate-adjusted DID = 33.3 min (95% CI 5.2, 61.5); *p* = 0.020], and total minutes spent at the park in the past 30 days [covariate-adjusted DID = 573.0 min (95% CI 69.3, 1076.7); *p* = 0.026] as a result of park renovation (Table 3). Residents who reported an annual household income of less than $20,000 experienced no significant effects of park renovation on any park use measure.

#### Employment status

Residents who were not employed experienced a significant increase in minutes spent at the park on weekdays [covariate-adjusted DID = 41.6 min (95% CI 15.5, 67.6); *p* = 0.002], minutes spent at the park on weekends [covariate-adjusted DID = 29.8 min (95% CI 3.5, 56.2); *p* = 0.026], and total minutes spent at the park in the past 30 days [covariate-adjusted DID = 791.7 min (95% CI 213.3, 1370.2); *p* = 0.007] as a result of park renovation, while residents who were employed or self-employed experienced no significant effect of park renovation on any park use measure (Table 3).

#### Children in household

Finally, residents with one or more children in their household experienced a significant increase in minutes spent at the study park on weekdays [covariate-adjusted DID = 36.8 min (95% CI 13.6, 60.0); *p* = 0.002] and total minutes spent at the study park in the past 30 days [covariate-adjusted DID = 498.8 min (95% CI 41.3, 956.4); *p* = 0.033] as a result of park renovation (Table 3). Residents with no children experienced no significant effect of park renovation on any park use measure.

### Changes in park satisfaction

**Fig. 2:**
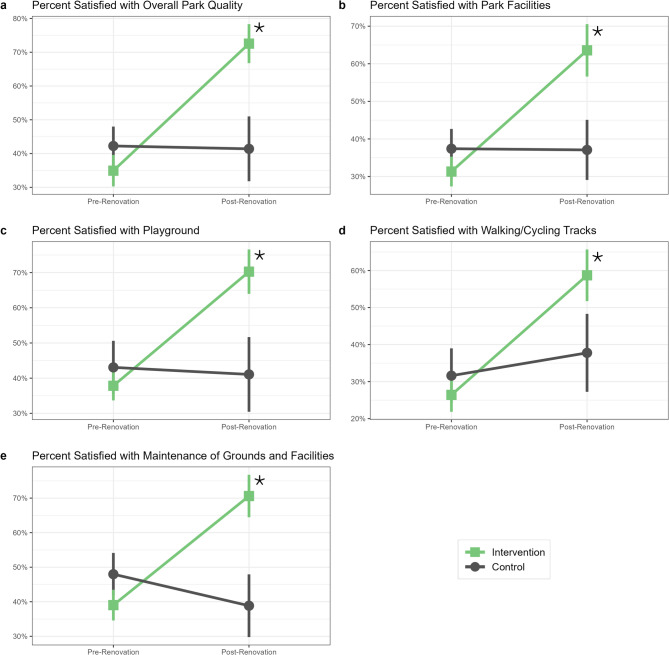
Mean park satisfaction measures among adult residents living near study parks before and after CPI park renovations. Means by intervention group (green square = intervention, grey circle = control) and 95% confidence intervals (vertical bars) were estimated using linear GEE models that included variables for time (pre-renovation vs. post-renovation), intervention group (renovated vs. control parks), and an interaction between time and intervention group (the DID estimator). An asterisk denotes a significant (*p* < 0.05) DID estimator after adjusting for age group, BMI category, annual household income, public housing, marital status, and children in household. *BMI* body mass index, *CPI* community parks initiative, *DID* difference-in-differences, *GEE* generalized estimating equations.

As shown in Table 4 and Fig. 2, park satisfaction across all measures increased over time in renovated park neighborhoods compared to control park neighborhoods. The largest improvements were observed in the percent of residents satisfied with overall park quality [covariate-adjusted DID = 38.4% (95% CI 25.2, 51.6); BH-corrected *p* < 0.001] and the maintenance of grounds and facilities [covariate-adjusted DID = 40.9% (95% CI 27.7, 54.1); BH-corrected *p* < 0.001]. Additionally, there were significantly larger increases in the percent of residents satisfied with the available park facilities [covariate-adjusted DID = 33.1% (95% CI 20.7, 45.6); BH-corrected *p* < 0.001], playgrounds [covariate-adjusted DID = 35.1% (95% CI 20.1, 50.1); BH-corrected *p* < 0.001], and walking/cycling tracks [covariate-adjusted DID = 27.3% (95% CI 12.5, 42.1); BH-corrected *p* = 0.001] among those living near renovated parks compared to those living near control parks.

**Table 4:** Changes in self-reported study park satisfaction among adult residents living in intervention vs. control park neighborhoods.

Study park satisfaction	Intervention park neighborhoods			Control park neighborhoods			Difference-in-differences		
	Pre-renovation^a^ (n = 545)	Post-renovation^a^ (n = 201)	Change (95% CI)^b^	Pre-renovation^a^ (n = 345)	Post-renovation^a^ (n = 129)	Change (95% CI)^b^	Unadjusted DID estimator (95% CI)^c^	Adjusted DID estimator (95% CI)^c,d^	*p*-value for adjusted DID Estimator^e^
Percent satisfied with overall park quality	34.9 (2.4)	72.5 (2.9)	37.6 (30.2, 45.0)	42.2 (2.9)	41.4 (4.9)	− 0.8 (− 12.0, 10.3)	38.5 (25.0, 51.9)	38.4 (25.2, 51.6)	** < 0.001**
Percent satisfied with park facilities	31.3 (2.0)	63.6 (3.6)	32.2 (24.2, 40.3)	37.4 (2.7)	37.1 (4.1)	− 0.3 (− 9.9, 9.3)	32.6 (20.1, 45.1)	33.1 (20.7, 45.6)	** < 0.001**
Percent satisfied with playground	37.8 (2.1)	70.3 (3.2)	32.4 (24.9, 40.0)	43.1 (3.9)	41.1 (5.4)	− 2.0 (− 15.0, 11.0)	34.4 (19.4, 49.5)	35.1 (20.1, 50.1)	** < 0.001**
Percent satisfied with walking/cycling tracks	26.4 (2.3)	58.7 (3.6)	32.3 (24.0, 40.7)	31.6 (3.8)	37.8 (5.4)	6.2 (− 6.7, 19.0)	26.1 (10.8, 41.5)	27.3 (12.5, 42.1)	**0.001**
Percent satisfied with the maintenance of the grounds and facilities	39.0 (2.3)	70.6 (3.1)	31.6 (24.0, 39.2)	48.0 (3.1)	38.8 (4.6)	− 9.1 (− 20.1, 1.8)	40.7 (27.4, 54.1)	40.9 (27.7, 54.1)	** < 0.001**

All estimates presented in table were pooled across GEE models fit to 25 imputed data sets. All GEE models included variables for time (pre-renovation vs. post-renovation), intervention group (renovated vs. control parks), and an interaction between time and intervention group (the DID estimator).

*BMI* body mass index, *CI* confidence interval, *DID* difference-in-differences, *GEE* generalized estimating equations, *SE* standard error.

^a^Unadjusted percent (SE).

^b^Unadjusted within-group change (post–pre).

^c^Difference in change in the given outcome measure in the intervention group minus the control group, as estimated by the interaction term between time * intervention group.

^d^Adjusted for age group, BMI category, annual household income, public housing, marital status, and children in household.

^e^Corrected using the Benjamini–Hochberg procedure to control the false discovery rate; significant (*p* < 0.05) *p*-values are bolded.

### Changes in park satisfaction stratified by sociodemographics

Table 5 demonstrates the varying effects of park renovation on changes in park satisfaction we observed across different sociodemographic groups, including: age, BMI, race/ethnicity, marital status, and the presence of children in households. We found no differences in the effects of park renovation on changes in park satisfaction by sex, annual household income, education, employment status, or public housing.

**Table 5:** Park satisfaction DID estimates stratified by age category, BMI category, race/ethnicity, marital status, and children in household.

	Overall park quality		Park facilities		Playground		Walking/cycling tracks		Maintenance of grounds and facilities	
	Adjusted DID estimator (95% CI)^a,b^	*p*-value^c^	Adjusted DID estimator (95% CI)^a,b^	*p*-value^c^	Adjusted DID estimator (95% CI)^a,b^	*p*-value^c^	Adjusted DID estimator (95% CI)^a,b^	*p*-value^c^	Adjusted DID estimator (95% CI)^a,b^	*p*-value^c^
Age category										
18–34 y	21.3 (0.5, 42.1)	**0.044**	33.0 (11.7, 54.3)	**0.002**	25.6 (2.7, 48.4)	**0.029**	14.4 (− 6.8, 35.6)	0.184	39.2 (17.4, 61.0)	** < 0.001**
35–49 y	59.5 (40.3, 78.7)	** < 0.001**	36.9 (17.5, 56.3)	** < 0.001**	48.0 (27.3, 68.7)	** < 0.001**	30.7 (9.1, 52.3)	**0.005**	51.8 (32.5, 71.1)	** < 0.001**
50–78 y	30.6 (6.1, 55.2)	**0.015**	24.6 (0.8, 48.4)	**0.043**	22.3 (− 3.5, 48.1)	0.091	36.2 (8.6, 63.7)	**0.010**	25.1 (2.3, 47.8)	**0.031**
BMI category										
Healthy (BMI < 25 kg/m^2^)	59.9 (37.0, 82.7)	** < 0.001**	39.5 (17.2, 61.7)	** 0.001**	46.3 (21.5, 71.2)	** < 0.001**	51.9 (30.6, 73.2)	** < 0.001**	64.6 (41.9, 87.3)	** < 0.001**
Overweight (BMI 25–29 kg/m^2^)	14.6 (− 9.7, 38.8)	0.239	16.7 (− 7.0, 40.5)	0.167	9.0 (− 15.9, 34.0)	0.477	6.7 (− 16.7, 30.0)	0.576	18.8 (− 7.0, 44.6)	0.154
Obese (BMI ≥ 30 kg/m^2^)	40.7 (18.4, 62.9)	** < 0.001**	40.9 (21.4, 60.5)	** < 0.001**	45.5 (25.3, 65.6)	** < 0.001**	27.2 (6.3, 48.2)	**0.011**	41.2 (21.2, 61.1)	** < 0.001**
Race/ethnicity										
Latino/a	33.9 (14.4, 53.4)	** 0.001**	31.7 (12.0, 51.4)	**0.002**	31.9 (11.4, 52.5)	**0.002**	14.3 (− 6.8, 35.4)	0.183	42.1 (23.7, 60.6)	** < 0.001**
Non-Latino/a black	47.2 (28.4, 66.1)	** < 0.001**	32.7 (15.2, 50.2)	** < 0.001**	46.3 (25.7, 66.9)	** < 0.001**	39.2 (16.4, 62.0)	** 0.001**	43.2 (22.9, 63.4)	** < 0.001**
Other/multiracial	33.8 (3.0, 64.5)	**0.031**	40.0 (9.8, 70.2)	**0.009**	24.5 (− 7.2, 56.3)	0.130	34.1 (6.3, 61.9)	**0.016**	35.7 (1.4, 70.1)	**0.042**
Marital status										
Never married	33.0 (14.1, 51.9)	** 0.001**	26.5 (8.6, 44.4)	**0.004**	23.5 (2.6, 44.3)	**0.028**	21.8 (3.2, 40.3)	**0.021**	40.2 (21.3, 59.0)	** < 0.001**
Married	39.6 (13.9, 65.3)	**0.003**	39.2 (16.4, 62.1)	**0.001**	41.8 (17.8, 65.8)	**0.001**	20.9 (− 4.1, 45.9)	0.101	50.8 (26.6, 75.0)	** < 0.001**
Divorced, separated, or widowed	52.1 (28.4, 75.8)	** < 0.001**	42.0 (17.6, 66.4)	**0.001**	58.3 (30.8, 85.8)	** < 0.001**	51.4 (21.9, 81.0)	**0.001**	31.4 (4.3, 58.5)	**0.023**
Children in household										
No children	28.1 (4.1, 52.0)	**0.022**	3.7 (− 20.3, 27.8)	0.760	9.3 (− 18.1, 36.8)	0.504	21.5 (− 5.0, 48.0)	0.111	19.7 (− 5.5, 45.0)	0.126
One or more children	41.5 (26.3, 56.6)	** < 0.001**	41.8 (28.3, 55.2)	** < 0.001**	41.9 (25.6, 58.2)	** < 0.001**	28.9 (13.3, 44.6)	** < 0.001**	48.0 (33.7, 62.2)	** < 0.001**

All estimates presented in table were pooled across GEE models fit to 25 imputed data sets. All GEE models included variables for time (pre-renovation vs. post-renovation), intervention group (renovated vs. control parks), and an interaction between time and intervention group (the DID estimator).

*BMI* body mass index, *CI* confidence interval, *DID* difference-in-differences, *GEE* generalized estimating equations.

^a^Difference in change in the given outcome measure in the intervention group minus the control group, as estimated by the interaction term between time * intervention group.

^b^Adjusted for age group, BMI category, annual household income, public housing, marital status, and children in household.

^c^Raw (uncorrected) *p*-value; significant (*p* < 0.05) *p*-values are bolded.

#### Age

In general, middle-aged residents (35–49 y) reported a larger increase in park satisfaction attributable to the CPI park renovation intervention compared to younger (18–34 y) and older (50–78 y) residents (Table 5). The largest difference between age groups occurred in satisfaction with overall park quality [18–34 y DID = 21.3% (95% CI 0.5, 42.1); *p* = 0.044); 35–49 y DID = 59.5% (95% CI 40.3, 78.7); *p* < 0.001; 50–78 y DID = 30.6% (95% CI 6.1, 55.2); *p* = 0.015)]. Individuals in the oldest age group (50–78 y) demonstrated a larger increase in satisfaction with walking/cycling tracks due to park renovation [covariate-adjusted DID = 36.2% (95% CI 8.6, 63.7); *p* = 0.010] compared to their younger counterparts [18–34 y DID = 14.4% (95% CI − 6.8, 35.6); *p* = 0.184; 35−49 y DID = 30.7% (95% CI 9.1, 52.3); *p* = 0.005].

#### BMI

Residents with healthy BMIs (< 25 kg/m^2^) experienced a larger increase in all park satisfaction measures as a result of park renovation compared to the higher BMI groups (Table 5). The largest difference between BMI groups was reported in satisfaction with walking/cycling tracks [healthy (BMI < 25 kg/m^2^) DID = 51.9% (95% CI 30.6, 73.2); *p* < 0.001; overweight (BMI 25–29 kg/m^2^) DID = 6.7% (95% CI − 16.7, 30.0); *p* = 0.576; obese (BMI ≥ 30 kg/m^2^) DID = 27.2% (95% CI 6.3, 48.2); *p* = 0.011].

#### Race/ethnicity

Non-Latino/a Black residents also generally experienced larger increases in park satisfaction measures attributable to park renovation compared to other racial/ethnic groups (Table 5). The largest difference between racial/ethnic groups was reported in satisfaction with park playgrounds [Latino/a DID = 31.9% (95% CI 11.4, 52.5); p = 0.002; non-Latino/a Black DID = 46.3% (95% CI 25.7, 66.9); *p* < 0.001; other/multiracial DID = 24.5% (95% CI − 7.2, 56.3); *p* = 0.130]. However, other/multiracial individuals had a larger increase in satisfaction with available park facilities [covariate-adjusted DID = 40.0% (95% CI 9.8, 70.2); *p* = 0.009] compared to other racial/ethnic groups [Latino/a DID = 31.7% (95% CI 12.0, 51.4); *p* = 0.002; non-Latino/a Black DID = 32.7% (95% CI 15.2, 50.2); *p* < 0.001].

#### Marital status

Divorced, separated, or widowed residents also generally experienced a larger increase in park satisfaction measures compared to never married and married groups (Table 5). The largest difference between groups occurred in satisfaction with walking/cycling tracks [never married DID = 21.8% (95% CI 3.2, 40.3); *p* = 0.021; married DID = 20.9% (95% CI − 4.1, 45.9); *p* = 0.101; divorced/separated/widowed DID = 51.4% (95% CI 21.9, 81.0); *p* = 0.001]. However, married individuals had a larger increase in satisfaction with the maintenance of park grounds and facilities [covariate-adjusted DID = 50.8% (95% CI 26.6, 75.0); *p* < 0.001] compared to never married [covariate-adjusted DID = 40.2% (95% CI 21.3, 59.0); *p* < 0.001] and divorced/separated/widowed individuals [covariate-adjusted DID = 31.4% (95% CI 4.3, 58.5); *p* = 0.023].

#### Children in household

Finally, residents with one or more children in their household experienced a significant increase in all park satisfaction measures as a result of park renovation, with the largest increase in the percent satisfied with maintenance of park grounds and facilities [covariate-adjusted DID = 48.0% (95% CI 33.7, 62.2); *p* < 0.001] (Table 5). In contrast, residents with no children only experienced a significant increase in satisfaction with overall park quality as a result of park renovation [covariate-adjusted DID = 28.1% (95% CI 4.1, 52.0); *p* = 0.022].

### Sensitivity analysis

Excluding 41 individual residents who completed post-renovation park use and satisfaction surveys during or after March 2020 did not result in substantial changes to the overall associations between CPI park renovations and park use outcomes (Supplementary Table 2) or park satisfaction outcomes (Supplementary Table 3). For the outcome of total minutes spent at the park in the past month, the previously statistically significant positive DID effect (BH-corrected *p* < 0.05) became marginally significant (BH-corrected *p* = 0.056), likely due to a reduction in statistical power.

## Discussion

In one of the largest quasi-experimental studies of its kind, we found that park renovations as part of the CPI were associated with neighborhood-level increases in park use and satisfaction with park quality and amenities in low-income NYC neighborhoods. Specifically, CPI led to a significantly larger increase in the usual length of time spent at study parks on weekdays and the total time spent at the study park in the past month among residents living near renovated parks compared to those living near control parks. CPI also led to increased satisfaction in overall park quality, available park facilities, playground amenities, walking/cycling tracks, and maintenance of park grounds and facilities among residents living near renovated parks. These findings highlight the important role of high-quality urban park spaces in promoting positive perceptions of parks and increased park use.

Our findings on the effects of park renovations on changes in park use align with and build upon evidence from previous intervention studies. A recent systematic review^21^ identified nine published studies that evaluated either park renovations or new implementation of urban greenspaces that included at least one outcome on park use^13,43–50^. All nine studies identified at least one significant positive effect on park use. Two additional natural experiment studies published after the systematic review and conducted in disadvantaged urban areas in Manchester, United Kingdom and Melbourne, Australia also identified significant effects of park renovations on the total number of observed park users^23,24^. We identified a similar significant effect of CPI park renovations on the total number of observed park users in a previous publication^22^. Only two other studies evaluated the implementation of renovations at 10 or more parks^44,47^.

This study makes a significant and novel contribution to the literature on neighborhood-level effects of park renovations on park use patterns and park perceptions, as it is one of the first and largest studies tracking changes in self-reported park use and satisfaction measures through repeated cross-sectional surveys administered to residents living near study parks. Unlike our study, most prior intervention studies have relied solely on systematic observations of park users or intercept surveys of park visitors, and therefore provide evidence of the effects of park renovations on park use at the park or park visitor level. We identified only three other studies that reported on neighborhood-level changes in self-reported park use, which showed null or positive associations between park renovations and various self-reported measures of park use^43,45,50^. Furthermore, while there is an expansive literature on social, demographic, contextual, and park feature-based determinants of park satisfaction^14,25–28^, we identified no other published study that has reported on changes in park satisfaction attributable to park improvements using quasi-experimental methods with matched control groups.

In exploratory analyses, we uncovered differences in the effects of park renovations on changes in park use and satisfaction across different BMI groups. Specifically, we found that park renovations were associated with more frequent park use among residents with an overweight BMI (25–29 kg/m^2^), and greater satisfaction among residents with a healthy BMI (< 25 kg/m^2^). Cross-sectional studies have linked better access to park spaces and higher perceived park quality and cleanliness with lower BMI^51–53^. Parks that are equipped with a variety of physical activity-promoting features^54^ may be appealing for individuals who are not already regularly physically active, which could provide one explanation for the larger increase in frequency of renovated park use that we observed in the overweight BMI group. Furthermore, Bai et al.^51^ showed that individuals with lower BMI were more likely to perceive parks as a benefit, which may offer an explanation for the larger increase in satisfaction with renovated parks that we observed among individuals with healthy BMIs in our study.

We also observed distinct differences in the effects of park renovations on park use and satisfaction in different racial/ethnic groups. We found that park renovations were generally associated with larger increases in park use among Latino/as and larger increases in park satisfaction among non-Latino/a Blacks. Latino/as have reported engaging in a variety of activities in public parks, such as socializing, using playgrounds, barbequing/picnicking, walking, and playing group sports^55–57^. Qualitative research has highlighted the strong connection Latino/as have with parks, perceiving them as cherished spaces for socialization and leisure with family and friends, akin to Mexican *plazas*^58^. Previous research from our team revealed that Latino/as living near recently renovated parks in NYC used these parks more frequently than any other racial/ethnic group and showed the strongest association between frequent park use and physical activity^15^. The variety of amenities provided by CPI renovations, and the strong communal connection Latino/as have with public park spaces, may have been drivers of the large increase in park use among Latino/as that we observed in this study. Conversely, our earlier published findings showed that Black NYC residents were using recently renovated parks less frequently than their Latino/a counterparts^15^. Black individuals have also previously cited personal safety concerns as a significant barrier to park use^57^, a perception that may not have been fully shifted by CPI, potentially explaining why Black residents in our study experienced the largest improvements in park perceptions, but did not experience significant changes in park use as a result of park renovations.

Across all measures, residents with one or more children in their household reported a larger increase in park use and satisfaction as a result of park renovations compared to those with no children. Nearly all intervention parks in the study received major upgrades to kid-friendly amenities, including playgrounds, splash pads, and other interactive play equipment, which likely drove these increases. Individuals without children who responded to study surveys did not experience a significant increase in any park use measure and only experienced a small increase in satisfaction with overall park quality as a result of park renovations. These findings are aligned with other intervention studies that have generally shown park renovations to be associated with a larger increase in the observed number of child and adult park users compared to adolescent/teen and senior adult park users^12,13,48,50^. Additional interventions beyond park renovations are likely needed to engage adults without children at parks, such as adult-friendly programming like group exercise activities and community social events.

Finally, we also observed a stronger effect of park renovations on changes in park satisfaction among middle-aged adults (35–49 y) and divorced, separated or widowed adults compared to other age and marital status groups, except in the case of satisfaction with walking/cycling tracks, where older adults (50–78 y) experienced the largest increase attributable to park renovation. While we did control the GEE models stratified by age and marital status for children in household, the fact that 35–49 y old and divorced/separated/widowed adults are more likely to have children compared to other groups may offer one explanation for these findings. Interestingly, we found that adults ≥ 50 y experienced the largest increase in satisfaction with walking/cycling tracks attributable to park renovations, which could suggest that older adults prefer walking/cycling tracks at their local parks. Previous studies have shown that older adults tend to use public parks less than their younger counterparts^15,59,60^, so walking/cycling tracks may be an important feature to support increased park use among this group. Studies have also shown that risk of injury can be a barrier to older adults participating in physical activity at parks^61^, so dedicated walking paths that are safe, well-maintained, and accessible may be particularly appealing for older adults who prefer walking as a means of physical activity^62^.

Our analysis of the effects of park renovations on changes in park use and satisfaction among various sociodemographic subgroups indicates that not all groups benefited from CPI park renovations in the same way. These findings can assist urban planners and policymakers in designing future park renovation and programming initiatives to better address the needs and preferences of diverse groups. For instance, non-Latino/a Black residents experienced the largest increase in park satisfaction due to the renovations, but this did not correspond to a significant rise in park use. Improving park use for this group may require addressing external factors such as neighborhood safety^57^. As another example, neighborhood residents without children did not experience an increase in park use and only showed a modest improvement in satisfaction with overall park quality attributable to park renovations. While the CPI intervention aimed to make renovated park spaces more inclusive for all ages by including features such as shaded seating areas, walking paths, and fitness equipment, these elements may not have been sufficient to encourage more park use among adults without children. Policy and programming initiatives beyond the physical features of parks may be necessary to enhance park use among these groups, including addressing social and contextual barriers to park use. To maximize the impact of park improvements on the health of urban communities, future initiatives may need to adopt multipronged, community-driven approaches that combine physical redesign, park programming, and community social interventions in ways that prioritize the unique needs and preferences of the communities they serve.

This study has several limitations. First, the survey instruments we used captured self-reported and subjective measures of park use and park satisfaction, which may be open to bias. Second, the surveyed residents at each wave of data collection represented convenience samples. While we showed that pre- and post-renovation samples were similar to each other, they might not have fully reflected the underlying populations in the study catchment areas, thereby limiting the generalizability of the study findings. However, although surveyed residents may not reflect truly random and population-representative samples of the neighborhoods surrounding study parks, the consistent manner of sampling we used allowed for a fair comparison of the pre- and post-renovation outcomes in both treatment groups. Finally, only one round of pre-renovation data was collected, which limited our ability to empirically evaluate the parallel trends assumption for causal inference using DID. To address this, we used five-year estimates at the census-block level from the American Community Survey to examine trends over time in the sociodemographic composition (e.g., % Black, % Latino/a, % in poverty, % with a Bachelor’s degree or higher, % disabled, % over 50 years of age) within intervention and control neighborhoods at three time points prior to the CPI intervention (2013, 2015, and 2017)^63^. We found no differences in the rate of change in the sociodemographic composition of intervention and control neighborhoods prior to the study period, which suggests that the parallel trends assumption may be reasonably met.

In conclusion, CPI park renovations led to significant neighborhood-level increases in park use and satisfaction, particularly time spent at the study park on weekdays, total time spent at the study park in the past month, satisfaction with overall park quality, and satisfaction with the maintenance of park grounds and facilities. We identified notable differences in the effects of CPI within different subgroups, revealing stronger effects of CPI on park use among Latino/as, residents with children, and residents with an overweight BMI (25–29 kg/m^2^). We also found stronger effects of CPI on park satisfaction among non-Latino/a Blacks, middle-aged and older adults, residents with children, and residents with a healthy BMI (< 25 kg/m^2^). These positive outcomes highlight the success of CPI as a large-scale park redesign and renovation effort, providing valuable insights for policymakers and urban planners striving to enhance public health and well-being through improved urban green spaces.

## Electronic supplementary material

Below is the link to the electronic supplementary material.

Supplementary Material 1

## Data Availability

The data needed to reproduce the findings from this study are available from the corresponding author upon reasonable request.
