# Correction: Park use patterns and park satisfaction before and after citywide park renovations in low-income New York City neighborhoods

**DOI:** 10.1038/s41598-026-37364-7

**Published:** 2026-02-24

**Authors:** Rachel L. Thompson, Katarzyna E. Wyka, Kelly R. Evenson, Lorna E. Thorpe, Glen D. Johnson, Brian T. Pavilonis, Terry T.-K. Huang

**Affiliations:** 1 Center for Systems and Community Design, Graduate School of Public Health and Health Policy, City University of New York (CUNY), New York, NY USA; 2 NYU-CUNY Prevention Research Center, New York, NY USA; 3 Department of Environmental, Occupational, and Geospatial Health Sciences, Graduate School of Public Health and Health Policy, City University of New York (CUNY), New York, NY USA; 4 Department of Epidemiology, Gillings School of Global Public Health, University of North Carolina–Chapel Hill, Chapel Hill, NC USA; 5 Department of Population Health, Grossman School of Medicine, New York University (NYU), New York, NY USA

Correction to: *Scientific Reports* 10.1038/s41598-025-07264-3, published online 02 July 2025

The original version of this Article contained errors in Table 1 and Supplementary Table S1, where a coding error resulted in incorrect mean and standard deviation values for the continuous BMI measures. It did not affect the BMI category variable, which was used in adjusted and stratified models.

The original Table 1 and accompanying legend appear below.

**Table 1:** Sociodemographic characteristics of sampled residents living within 0.3 miles of an intervention or control park, pre- and post-renovation.

	Overall sample			Pre-renovation by group			Post-renovation by group		
	Pre-renovation N = 890^a^	Post-renovation N = 330^a^	*p*-value^b^	Intervention N = 545^a^	Control N = 345^a^	*p*-value^b^	Intervention N = 201^a^	Control N = 129^a^	*p*-value^b^
Sex			0.300			0.120			0.400
Female	715 (81%)	267 (83%)		428 (79%)	287 (83%)		159 (82%)	108 (86%)	
Male	172 (19%)	53 (17%)		114 (21%)	58 (17%)		35 (18%)	18 (14%)	
(Missing)	3	10		3	0		7	3	
Age	38 (12)	41 (13)	** < 0.001**	38 (13)	38 (11)	0.600	41 (12)	42 (14)	0.800
Age category			**0.002**			0.500			**0.070**
18–34 y	394 (45%)	104 (34%)		241 (45%)	153 (45%)		59 (32%)	45 (37%)	
35–49 y	305 (35%)	125 (41%)		179 (34%)	126 (37%)		85 (46%)	40 (33%)	
50–78 y	172 (20%)	79 (26%)		110 (21%)	62 (18%)		42 (23%)	37 (30%)	
(Missing)	19	22		15	4		15	7	
Body mass index (BMI)	31 (31)	30 (8)	0.400	31 (40)	30 (7)	0.700	30 (8)	30 (7)	0.800
BMI category			**0.077**			**0.060**			0.400
Healthy (BMI < 25 kg/m^2^)	206 (24%)	89 (30%)		132 (26%)	74 (22%)		58 (33%)	31 (26%)	
Overweight (BMI 25–29 kg/m^2^)	269 (32%)	78 (27%)		170 (33%)	99 (29%)		43 (25%)	35 (30%)	
Obese (BMI ≥ 30 kg/m^2^)	376 (44%)	125 (43%)		209 (41%)	167 (49%)		73 (42%)	52 (44%)	
(Missing)	39	38		34	5		27	11	
Race/ethnicity			0.400			> 0.900			0.500
Latino/a	408 (47%)	130 (42%)		247 (47%)	161 (47%)		74 (40%)	56 (46%)	
Non-Latino/a Black	326 (37%)	123 (40%)		198 (38%)	128 (37%)		79 (43%)	44 (36%)	
Other or multiracial	136 (16%)	53 (17%)		83 (16%)	53 (15%)		32 (17%)	21 (17%)	
(Missing)	20	24		17	3		16	8	
Annual household income			0.300			0.300			**0.082**
$20,000 or more	410 (49%)	131 (45%)		258 (50%)	152 (47%)		85 (50%)	46 (39%)	
Less than $20,000	426 (51%)	157 (55%)		253 (50%)	173 (53%)		86 (50%)	71 (61%)	
(Missing)	54	42		34	20		30	12	
Education			0.700			0.500			0.800
High school graduate or less	432 (50%)	134 (49%)		266 (51%)	166 (48%)		76 (48%)	58 (50%)	
Some college or more	435 (50%)	142 (51%)		258 (49%)	177 (52%)		83 (52%)	59 (50%)	
(Missing)	23	54		21	2		42	12	
Employment status			0.300			0.200			0.140
Employed or self-employed	453 (51%)	157 (48%)		268 (50%)	185 (54%)		102 (51%)	55 (43%)	
Not employed	429 (49%)	172 (52%)		272 (50%)	157 (46%)		98 (49%)	74 (57%)	
(Missing)	8	1		5	3		1	0	
Public housing			0.600			** < 0.001**			**0.049**
Non-NYCHA resident	447 (50%)	157 (49%)		299 (55%)	148 (43%)		104 (53%)	53 (42%)	
NYCHA resident	440 (50%)	164 (51%)		243 (45%)	197 (57%)		91 (47%)	73 (58%)	
(Missing)	3	9		3	0		6	3	
Marital status			0.600			**0.032**			0.900
Never married	446 (50%)	175 (53%)		283 (52%)	163 (48%)		107 (54%)	68 (53%)	
Married	265 (30%)	89 (27%)		145 (27%)	120 (35%)		55 (28%)	34 (26%)	
Divorced, separated, or widowed	173 (20%)	64 (20%)		113 (21%)	60 (17%)		37 (19%)	27 (21%)	
(Missing)	6	2		4	2		2	0	
Children in household			0.400			**0.002**			0.400
No children	190 (22%)	79 (24%)		135 (25%)	55 (16%)		45 (22%)	34 (27%)	
One or more children	686 (78%)	250 (76%)		402 (75%)	284 (84%)		156 (78%)	94 (73%)	
(Missing)	14	1		8	6		0	1	

*BMI* body mass index, *NYCHA* New York City housing authority, *SD* standard deviation.

^a^n (%); Mean (SD).

^b^Pearson’s Chi-squared test; Welch Two Sample t-test; significant (*p* < 0.05) and marginally significant (0.05 < *p* < 0.1) *p*-values are bolded.

Supplementary Information Table S1 contained the same error. The original Table S1 and accompanying legend appear below.

**Table S1:** Sociodemographic characteristics of survey respondents pre- and post-renovation using multiply imputed data.

	Overall sample			Pre-renovation by group			Post-renovation by group		
	Pre-renovation N = 890^a^	Post-renovation N = 330^a^	*p* value^b^	Intervention N = 545^a^	Control N = 345^a^	*p* value^b^	Intervention N = 201^a^	Control N = 129^a^	*p* value^b^
Sex (1.1% Imputed)			0.200			0.130			0.400
Female	718 (81%)	277 (84%)		431 (79%)	287 (83%)		166 (83%)	111 (86%)	
Male	172 (19%)	53 (16%)		114 (21%)	58 (17%)		35 (17%)	18 (14%)	
Age (3.4% imputed)	38 (12)	41 (13)	**<0.001**	38 (13)	38 (11)	0.600	41 (12)	41 (14)	0.900
Age category			**0.005**			0.700			**0.063**
18–34y	395 (44%)	113 (34%)		242 (44%)	153 (44%)		64 (32%)	49 (38%)	
35–49y	322 (36%)	136 (41%)		193 (35%)	129 (37%)		93 (46%)	43 (33%)	
50–78y	173 (19%)	81 (25%)		110 (20%)	63 (18%)		44 (22%)	37 (29%)	
Body mass index (BMI) (6.3% imputed)	31 (30)	30 (7)	0.500	31 (38)	30 (7)	0.700	30 (8)	30 (7)	>0.900
BMI category			0.200			**0.054**			0.300
Healthy (BMI <25 kg/m^2^)	211 (24%)	91 (28%)		136 (25%)	75 (22%)		60 (30%)	31 (24%)	
Overweight (BMI 25-29 kg/m^2^)	288 (32%)	92 (28%)		187 (34%)	101 (29%)		51 (25%)	41 (32%)	
Obese (BMI ≥30 kg/m^2^)	391 (44%)	147 (45%)		222 (41%)	169 (49%)		90 (45%)	57 (44%)	
Race/ethnicity (3.6% imputed)			0.300			>0.900			0.500
Latino/a	417 (47%)	139 (42%)		256 (47%)	161 (47%)		80 (40%)	59 (46%)	
Non-Latino/a black	336 (38%)	138 (42%)		206 (38%)	130 (38%)		89 (44%)	49 (38%)	
Other or multiracial	137 (15%)	53 (16%)		83 (15%)	54 (16%)		32 (16%)	21 (16%)	
Annual household income (7.9% imputed)			0.200			0.300			**0.039**
$20,000 or more	429 (48%)	146 (44%)		271 (50%)	158 (46%)		98 (49%)	48 (37%)	
Less than $20,000	461 (52%)	184 (56%)		274 (50%)	187 (54%)		103 (51%)	81 (63%)	
Education (6.3% imputed)			0.700			0.400			0.800
High school graduate or less	446 (50%)	161 (49%)		279 (51%)	167 (48%)		99 (49%)	62 (48%)	
Some college or more	444 (50%)	169 (51%)		266 (49%)	178 (52%)		102 (51%)	67 (52%)	
Employment status (0.7% imputed)			0.300			0.200			0.130
Employed or self-employed	455 (51%)	158 (48%)		269 (49%)	186 (54%)		103 (51%)	55 (43%)	
Not employed	435 (49%)	172 (52%)		276 (51%)	159 (46%)		98 (49%)	74 (57%)	
Public housing (1.0% imputed)			0.800			**<0.001**			**0.049**
Non-NYCHA resident	448 (50%)	163 (49%)		300 (55%)	148 (43%)		108 (54%)	55 (43%)	
NYCHA resident	442 (50%)	167 (51%)		245 (45%)	197 (57%)		93 (46%)	74 (57%)	
Marital status (0.7% imputed)			0.500			**0.033**			0.900
Never married	448 (50%)	177 (54%)		284 (52%)	164 (48%)		109 (54%)	68 (53%)	
Married	268 (30%)	89 (27%)		147 (27%)	121 (35%)		55 (27%)	34 (26%)	
Divorced, separated, or widowed	174 (20%)	64 (19%)		114 (21%)	60 (17%)		37 (18%)	27 (21%)	
Children in household (1.2% imputed)			0.400			**0.003**			0.300
No children	195 (22%)	80 (24%)		137 (25%)	58 (17%)		45 (22%)	35 (27%)	
One or more children	695 (78%)	250 (76%)		408 (75%)	287 (83%)		156 (78%)	94 (73%)	

^a^n (%); Mean (SD)

^b^Pearson's chi-squared test; Welch two sample *t* test; significant (*p* < 0.05) and marginally significant (0.05 < *p* < 0.1) p-values are bolded

This table summarizes all imputed and non-imputed sociodemographic characteristics, such that for imputed cells the most commonly imputed value across the 25 imputed datasets was used.

*BMI* body mass index, *NYCHA* New York City Housing Authority, *SD* standard deviation

As the result, in the Results section, under the subheading ‘Sociodemographic characteristics of residents living near study parks’,

“There were marginally significant differences in the composition of the intervention and control samples by BMI category (pre-renovation only), age category (post-renovation only), and annual household income (post-renovation only).”

now reads:

“There were marginally significant differences in the composition of the intervention and control samples by BMI category and continuous BMI (pre-renovation only), age category (post-renovation only), and annual household income (post-renovation only).”

Additionally, Figures 1 and 2 legends incorrectly implied that the presented means and 95% confidence intervals were adjusted for covariates. The means and 95% confidence intervals in the figures were unadjusted. However, the asterisks denoting statistical significance were reflective of DID estimators that were adjusted for covariates.

As a result, in Figure 1 legend,

“Mean park use measures among adult residents living near study parks before and after CPI park renovations. Means by intervention group (green square = intervention, grey circle = control) and 95% confidence intervals (vertical bars) were estimated using linear GEE models that included variables for time (pre-renovation vs. post-renovation), intervention group (renovated vs. control parks), and an interaction between time and intervention group (the DID estimator), and additionally adjusted for age group, BMI category, annual household income, public housing, marital status, and children in household. An asterisk denotes a significant (*p* < 0.05) DID estimator. *BMI* body mass index, *CPI* community parks initiative, *DID* difference-in-differences, *GEE* generalized estimating equations.”

now reads:

“Mean park use measures among adult residents living near study parks before and after CPI park renovations. Means by intervention group (green square = intervention, grey circle = control) and 95% confidence intervals (vertical bars) were estimated using linear GEE models that included variables for time (pre-renovation vs. post-renovation), intervention group (renovated vs. control parks), and an interaction between time and intervention group (the DID estimator). An asterisk denotes a significant (*p* < 0.05) DID estimator after adjusting for age group, BMI category, annual household income, public housing, marital status, and children in household. BMI body mass index, CPI community parks initiative, DID difference-in-differences, GEE generalized estimating equations.”

In Figure 2 legend,

“Mean park satisfaction measures among adult residents living near study parks before and after CPI park renovations. Means by intervention group (green square = intervention, grey circle = control) and 95% confidence intervals (vertical bars) were estimated using linear GEE models that included variables for time (pre-renovation vs. post-renovation), intervention group (renovated vs. control parks), and an interaction between time and intervention group (the DID estimator), and additionally adjusted for age group, BMI category, annual household income, public housing, marital status, and children in household. An asterisk denotes a significant (*p* < 0.05) DID estimator. *BMI* body mass index, *CPI* community parks initiative, *DID* difference-in-differences, *GEE* generalized estimating equations.”

now reads:

“Mean park satisfaction measures among adult residents living near study parks before and after CPI park renovations. Means by intervention group (green square = intervention, grey circle = control) and 95% confidence intervals (vertical bars) were estimated using linear GEE models that included variables for time (pre-renovation vs. post-renovation), intervention group (renovated vs. control parks), and an interaction between time and intervention group (the DID estimator). An asterisk denotes a significant (*p* < 0.05) DID estimator after adjusting for age group, BMI category, annual household income, public housing, marital status, and children in household. *BMI* body mass index, *CPI* community parks initiative, *DID* difference-in-differences, *GEE* generalized estimating equations.”

The original Article and accompanying Supplementary Information file have been corrected.

## Supplementary Information

Supplementary Information.

