# Supplementary material for: Park use patterns and park satisfaction before and after citywide park renovations in low-income New York City neighborhoods
Source: Sci Rep. 2025 Jul 2;15:23036. doi: 10.1038/s41598-025-07264-3 (PMC12217957; doi:10.1038/s41598-025-07264-3)
Supplement: Supplementary file 1 — Supplementary Material 1 [file 41598_2025_7264_MOESM1_ESM.docx]

**Supplementary Table 1. Sociodemographic characteristics of survey respondents pre- and post-renovation using multiply imputed data**

|  | **Overall Sample** | | | **Pre-Renovation by Group** | | | **Post-Renovation by Group** | | |
| --- | --- | --- | --- | --- | --- | --- | --- | --- | --- |
|  | **Pre-Renovation**  N = 890*^1^* | **Post-Renovation**  N = 330*^1^* | **p-value***^2^* | **Intervention**  N = 545*^1^* | **Control**  N = 345*^1^* | **p-value***^2^* | **Intervention**  N = 201*^1^* | **Control**  N = 129*^1^* | **p-value***^2^* |
| **Sex** *(1.1% Imputed)* |  |  | 0.200 |  |  | 0.130 |  |  | 0.400 |
| Female | 718 (81%) | 277 (84%) |  | 431 (79%) | 287 (83%) |  | 166 (83%) | 111 (86%) |  |
| Male | 172 (19%) | 53 (16%) |  | 114 (21%) | 58 (17%) |  | 35 (17%) | 18 (14%) |  |
| **Age** *(3.4% Imputed)* | 38 (12) | 41 (13) | **<0.001** | 38 (13) | 38 (11) | 0.600 | 41 (12) | 41 (14) | 0.900 |
| **Age Category** |  |  | **0.005** |  |  | 0.700 |  |  | **0.063** |
| 18-34y | 395 (44%) | 113 (34%) |  | 242 (44%) | 153 (44%) |  | 64 (32%) | 49 (38%) |  |
| 35-49y | 322 (36%) | 136 (41%) |  | 193 (35%) | 129 (37%) |  | 93 (46%) | 43 (33%) |  |
| 50-78y | 173 (19%) | 81 (25%) |  | 110 (20%) | 63 (18%) |  | 44 (22%) | 37 (29%) |  |
| **Body Mass Index (BMI)** *(6.3% Imputed)* | 30 (7) | 30 (7) | 0.700 | 29 (7) | 30 (7) | **0.057** | 30 (8) | 30 (7) | >0.900 |
| **BMI Category** |  |  | 0.200 |  |  | **0.054** |  |  | 0.300 |
| Healthy (BMI <25 kg/m^2^) | 211 (24%) | 91 (28%) |  | 136 (25%) | 75 (22%) |  | 60 (30%) | 31 (24%) |  |
| Overweight (BMI 25-29 kg/m^2^) | 288 (32%) | 92 (28%) |  | 187 (34%) | 101 (29%) |  | 51 (25%) | 41 (32%) |  |
| Obese (BMI ≥30 kg/m^2^) | 391 (44%) | 147 (45%) |  | 222 (41%) | 169 (49%) |  | 90 (45%) | 57 (44%) |  |
| **Race/Ethnicity** *(3.6% Imputed)* |  |  | 0.300 |  |  | >0.900 |  |  | 0.500 |
| Latino/a | 417 (47%) | 139 (42%) |  | 256 (47%) | 161 (47%) |  | 80 (40%) | 59 (46%) |  |
| Non-Latino/a Black | 336 (38%) | 138 (42%) |  | 206 (38%) | 130 (38%) |  | 89 (44%) | 49 (38%) |  |
| Other or multiracial | 137 (15%) | 53 (16%) |  | 83 (15%) | 54 (16%) |  | 32 (16%) | 21 (16%) |  |
| **Annual Household Income** *(7.9% Imputed)* |  |  | 0.200 |  |  | 0.300 |  |  | **0.039** |
| $20,000 or more | 429 (48%) | 146 (44%) |  | 271 (50%) | 158 (46%) |  | 98 (49%) | 48 (37%) |  |
| Less than $20,000 | 461 (52%) | 184 (56%) |  | 274 (50%) | 187 (54%) |  | 103 (51%) | 81 (63%) |  |
| **Education** *(6.3% Imputed)* |  |  | 0.700 |  |  | 0.400 |  |  | 0.800 |
| High school graduate or less | 446 (50%) | 161 (49%) |  | 279 (51%) | 167 (48%) |  | 99 (49%) | 62 (48%) |  |
| Some college or more | 444 (50%) | 169 (51%) |  | 266 (49%) | 178 (52%) |  | 102 (51%) | 67 (52%) |  |
| **Employment Status** *(0.7% Imputed)* |  |  | 0.300 |  |  | 0.200 |  |  | 0.130 |
| Employed or self-employed | 455 (51%) | 158 (48%) |  | 269 (49%) | 186 (54%) |  | 103 (51%) | 55 (43%) |  |
| Not employed | 435 (49%) | 172 (52%) |  | 276 (51%) | 159 (46%) |  | 98 (49%) | 74 (57%) |  |
| **Public Housing** *(1.0% Imputed)* |  |  | 0.800 |  |  | **<0.001** |  |  | **0.049** |
| Non-NYCHA resident | 448 (50%) | 163 (49%) |  | 300 (55%) | 148 (43%) |  | 108 (54%) | 55 (43%) |  |
| NYCHA resident | 442 (50%) | 167 (51%) |  | 245 (45%) | 197 (57%) |  | 93 (46%) | 74 (57%) |  |
| **Marital Status** *(0.7% Imputed)* |  |  | 0.500 |  |  | **0.033** |  |  | 0.900 |
| Never married | 448 (50%) | 177 (54%) |  | 284 (52%) | 164 (48%) |  | 109 (54%) | 68 (53%) |  |
| Married | 268 (30%) | 89 (27%) |  | 147 (27%) | 121 (35%) |  | 55 (27%) | 34 (26%) |  |
| Divorced, separated, or widowed | 174 (20%) | 64 (19%) |  | 114 (21%) | 60 (17%) |  | 37 (18%) | 27 (21%) |  |
| **Children in Household** *(1.2% Imputed)* |  |  | 0.400 |  |  | **0.003** |  |  | 0.300 |
| No children | 195 (22%) | 80 (24%) |  | 137 (25%) | 58 (17%) |  | 45 (22%) | 35 (27%) |  |
| One or more children | 695 (78%) | 250 (76%) |  | 408 (75%) | 287 (83%) |  | 156 (78%) | 94 (73%) |  |
| *^1^*n (%); Mean (SD) | | | | | | | | | |
| *^2^*Pearson's Chi-squared test; Welch Two Sample t-test; significant (p<0.05) and marginally significant (0.05<p< 0.1) p-values are bolded  This table summarizes all imputed and non-imputed sociodemographic characteristics, such that for imputed cells the most commonly imputed value across the 25 imputed datasets was used.  Abbreviations – BMI: body mass index; NYCHA: New York City Housing Authority; SD: standard deviation | | | | | | | | | |

**Supplementary Table 2. Changes in self-reported past-month study park use among adult residents living in intervention vs. control park neighborhoods after excluding those with surveys completed during and after March 2020 (COVID-19 pandemic)**

|  | **Intervention Park Neighborhoods** | | | **Control Park Neighborhoods** | | | **Difference-in-Differences** | | |
| --- | --- | --- | --- | --- | --- | --- | --- | --- | --- |
| **Past-Month Study Park Use** | **Pre-Renovation**^1^  **(n=545)** | **Post-Renovation**^1^  **(n=169)** | **Change**  **(95% CI)** ^2^ | **Pre-Renovation**^1^  **(n=345)** | **Post-Renovation**^1^  **(n=120)** | **Change**  **(95% CI)** ^2^ | **Unadjusted DID Estimator**  **(95% CI)^3^** | **Adjusted**  **DID Estimator (95% CI)^3,4^** | **p-value for Adjusted**  **DID Estimator^5^** |
| Days visited | 9.8 (0.6) | 11.6 (0.9) | 1.8 (-0.3, 4.0) | 8.4 (0.7) | 8.1 (1.0) | -0.3 (-2.6, 2.0) | 2.2 (-1.0, 5.3) | 2.1 (-1.0, 5.3) | 0.193 |
| Minutes spent at park on weekdays | 62.7 (3.8) | 75.0 (6.6) | 12.3 (-2.7, 27.2) | 64.5 (5.9) | 51.0 (4.9) | -13.6 (-28.7, 1.6) | 25.8 (4.5, 47.1) | 25.6 (4.8, 46.5) | **0.032** |
| Minutes spent at park on weekend days | 62.6 (3.5) | 70.6 (5.9) | 8.0 (-5.5, 21.6) | 59.3 (5.8) | 52.2 (6.5) | -7.1 (-24.1, 10.0) | 15.1 (-6.7, 36.9) | 14.1 (-7.1, 35.4) | 0.193 |
| Total minutes spent at park | 795.1 (79.9) | 1130.4 (148.5) | 335.4 (4.4, 666.3) | 697.7 (90.2) | 565.6 (105.1) | -132.0 (-403.8, 139.7) | 467.4 (39.2, 895.6) | 470.1 (38.8, 901.3) | **0.056** |
| Percent visiting park ≥once per month | 82.0 (1.6) | 84.1 (2.9) | 2.1 (-4.4, 8.7) | 79.1 (3.1) | 66.8 (5.0) | -12.3 (-23.8, -0.7) | 14.4 (1.1, 27.6) | 13.8 (0.4, 27.2) | **0.065** |
| Percent visiting park ≥30 minutes on a weekday | 59.5 (3.0) | 61.6 (4.2) | 2.1 (-8.1, 12.3) | 63.0 (4.8) | 48.6 (4.6) | -14.4 (-27.4, -1.3) | 16.5 (-0.1, 33.0) | 15.5 (-0.2, 31.2) | **0.068** |
| Percent visiting park ≥30 minutes on a weekend day | 57.1 (2.9) | 58.4 (4.0) | 1.4 (-8.3, 11.0) | 57.6 (4.9) | 42.3 (5.0) | -15.3 (-29.0, -1.6) | 16.6 (-0.1, 33.4) | 15.8 (-0.4, 32.0) | **0.068** |
| All estimates presented in table were pooled across GEE models fit to 25 imputed data sets. All GEE models included variables for time (pre-renovation vs. post-renovation), intervention group (renovated vs. control parks), and an interaction between time and intervention group (the DID estimator).  ^1^Unadjusted mean (SE) or percent (SE)  ^2^Unadjusted within-group change (post-pre)  ^3^Difference in change in the given outcome measure in the intervention group minus the control group, as estimated by the interaction term between time * intervention group  ^4^Adjusted for age group, BMI category, annual household income, public housing, marital status, and children in household  ^5^Corrected using the Benjamini-Hochberg procedure to control the false discovery rate; significant (p<0.05) and marginally significant (0.05<p< 0.1) p-values are bolded  Abbreviations – BMI: body mass index; CI: confidence interval; DID: Difference-in-Differences; GEE: generalized estimating equations; SE: standard error | | | | | | | | | |

**Supplementary Table 3. Changes in self-reported study park satisfaction among adult residents living in intervention vs. control park neighborhoods after excluding those with surveys completed during and after March 2020 (COVID-19 pandemic)**

|  | **Intervention Park Neighborhoods** | | | **Control Park Neighborhoods** | | | **Difference-in-Differences** | | |
| --- | --- | --- | --- | --- | --- | --- | --- | --- | --- |
| **Past-Month Study Park Satisfaction** | **Pre-Renovation**^1^  **(n=545)** | **Post-Renovation**^1^  **(n=169)** | **Change**  **(95% CI)** ^2^ | **Pre-Renovation**^1^  **(n=345)** | **Post-Renovation**^1^  **(n=120)** | **Change**  **(95% CI)** ^2^ | **Unadjusted DID Estimator**  **(95% CI)^3^** | **Adjusted**  **DID Estimator (95% CI)^3,4^** | **p-value for Adjusted**  **DID Estimator^5^** |
| Percent satisfied with overall park quality | 34.9 (2.4) | 70.8 (3.3) | 35.9 (27.9, 43.9) | 42.2 (2.9) | 41.1 (5.0) | -1.1 (-12.5, 10.3) | 37.0 (23.1, 51.0) | 37.0 (23.3, 50.7) | **<0.001** |
| Percent satisfied with park facilities | 31.3 (2.0) | 64.4 (4.0) | 33.0 (24.3, 41.8) | 37.4 (2.7) | 37.4 (4.1) | 0.00 (-9.7, 9.6) | 33.1 (20.1, 46.1) | 33.7 (20.7, 46.7) | **<0.001** |
| Percent satisfied with playground | 37.8 (2.1) | 71.2 (3.4) | 33.4 (25.5, 41.3) | 43.1 (3.9) | 40.7 (5.6) | -2.3 (-15.7, 11.0) | 35.7 (20.2, 51.3) | 36.5 (21.0, 51.9) | **<0.001** |
| Percent satisfied with walking/cycling tracks | 26.4 (2.3) | 59.9 (3.9) | 33.5 (24.6, 42.4) | 31.6 (3.8) | 37.1 (5.6) | 5.5 (-7.6, 18.7) | 28.0 (12.1, 43.9) | 28.9 (13.6, 44.2) | **0.001** |
| Percent satisfied with the maintenance of the grounds and facilities | 39.0 (2.3) | 69.8 (3.4) | 30.7 (22.7, 38.8) | 48.0 (3.1) | 38.4 (4.7) | -9.6 (-20.7, 1.6) | 40.3 (26.6, 54.0) | 40.6 (27.1, 54.2) | **<0.001** |
| All estimates presented in table were pooled across GEE models fit to 25 imputed data sets. All GEE models included variables for time (pre-renovation vs. post-renovation), intervention group (renovated vs. control parks), and an interaction between time and intervention group (the DID estimator).  ^1^Unadjusted percent (SE)  ^2^Unadjusted within-group change (post-pre)  ^3^Difference in change in the given outcome measure in the intervention group minus the control group, as estimated by the interaction term between time * intervention group  ^4^Adjusted for age group, BMI category, annual household income, public housing, marital status, and children in household  ^5^Corrected using the Benjamini-Hochberg procedure to control the false discovery rate; significant (p<0.05) p-values are bolded  Abbreviations – BMI: body mass index; CI: confidence interval; DID: Difference-in-Differences; GEE: generalized estimating equations; SE: standard error | | | | | | | | | |
